# TOTAL LYMPHOCYTE COUNT AND SERUM ALBUMIN AS PREDICTORS OF NUTRITIONAL RISK IN SURGICAL PATIENTS

**DOI:** 10.1590/S0102-67202015000300012

**Published:** 2015

**Authors:** Naruna Pereira ROCHA, Renata Costa FORTES

**Affiliations:** Clinical Nutrition Residency Program, Asa Norte Regional Hospital, Federal Department of Health, Brasília, DC, Brazil

**Keywords:** Hypoalbuminemia, Lymphocytes, Malnutrition, Ambulatory surgical procedures, Adult

## Abstract

**Background:**

Early detection of changes in nutritional status is important for a better approach to the surgical patient. There are several nutritional measures in clinical practice, but there is not a complete method for determining the nutritional status, so, health professionals should only choose the best method to use.

**Aim:**

To evaluate the total lymphocyte count and albumin as predictors of identification of nutritional risk in surgical patients.

**Methods:**

Prospective longitudinal study was conducted with 69 patients undergoing surgery of the gastrointestinal tract. The assessment of nutritional status was evaluated by objective methods (anthropometry and biochemical tests) and subjective methods (subjective global assessment).

**Results:**

All parameters used in the nutritional assessment detected a high prevalence of malnutrition, with the exception of BMI which detected only 7.2% (n=5). The albumin (p=0.01), the total lymphocytes count (p=0.02), the percentage of adequacy of skinfolds (p<0.002) and the subjective global assessment (p<0.001) proved to be useful as predictors of risk of postoperative complications, since the smaller the values of albumin and lymphocyte count and higher the score the subjective global assessment were higher risks of surgical complications.

**Conclusions:**

A high prevalence of malnutrition was found, except for BMI. The use of albumin and total lymphocyte count were good predictor for the risk of postoperative complications and when used with other methods of assessing the nutritional status, such as the subjective global assessment and the percentage of adequacy of skinfolds, can be useful for identification of nutritional risk and postoperative complications.

## Abstract (pt)

**Racional:**

A detecção precoce de alterações do estado nutricional é importante para melhor conduta do paciente cirúrgico. Existem várias medidas nutricionais na prática clínica, porém não há método completo para a determinação do estado nutricional cabendo ao profissional a escolha do melhor a ser utilizado.

**Objetivo:**

Avaliar se a contagem total de linfócitos e a albumina sérica atuam como preditores de risco nutricional em pacientes cirúrgicos.

**Métodos:**

Estudo longitudinal prospectivo com 69 pacientes submetidos à cirurgia do trato gastrointestinal. A avaliação do estado nutricional foi realizada por métodos objetivos (antropometria e exames bioquímicos) e subjetivos (avaliação subjetiva global).

**Resultados:**

Todos os parâmetros utilizados na avaliação nutricional detectaram elevada prevalência de desnutrição, com exceção do IMC que detectou apenas 7,2% (n=5). A albumina (p=0,01), a contagem total de linfócitos (p=0,02), o percentual de adequação da dobra cutânea tricipital (p<0,002) e a avaliação subjetiva global (p<0,001) mostraram-se úteis como preditoras do risco de complicações pós-operatórias, visto que quanto menores os valores de albumina e da contagem dos linfócitos e maior o escore da avaliação subjetiva global, maiores eram os riscos de complicações cirúrgicas.

**Conclusão:**

Elevada prevalência de desnutrição foi encontrada, com exceção IMC. A utilização da albumina e da contagem total dos linfócitos indicaram boa predição do risco de complicações pós-operatórias, que utilizadas em conjuto com a avaliação subjetiva global e o percentual de adequação da dobra cutânea tricipital, podem ser úteis para identificação de riscos nutricionais e complicações pós-operatórias.

## INTRODUCTION

Malnutrition has a high prevalence in the hospital environment, considering that approximately 40% of patients are already undernourished at the time of admission at the hospital, and about 75% have involuntary weight loss during hospitalization 20 .

Hospital malnutrition can affect negatively the clinical evolution of the patients because it can increase the time of hospitalization, the incidence of postoperative complications, such as infections and delay of the scarring process of wounds, and it can also increase the mortality rate 9 13 . A study conducted in 1936 had already showed a strong correlation between postoperative malnutrition and complications and mortality in patients after major surgeries in the abdominal cavity 21 .

In the clinical practice, there are several nutritional measures; however, there is no complete method to determine the nutritional status, and the disease or trauma can affect all of the measurements used in the nutritional assessment. It is important to observe that there is no method without at least one important limitation 25 .

The analytical parameters, such as the total lymphocytes count (TLC) and serum albumin, were often used as nutritional indexes combined or not with other parameters of nutritional status, and they proved to be valid and reliable for this purpose 17 . However, the laboratorial indicators used separately in the nutritional assessment failed to identify the individuals with a compromised nutritional status, because many conditions affect the serum values of these indicators 18 .

The early detection of malnutrition is important in order to implement an adequate nutritional therapy with the purpose of maintaining the nutritional status and avoiding the installation or the progression of malnutrition and its complications 12 .

Thus, it was important to evaluate if the total lymphocyte count and serum albumin could be used as predictors of nutritional risk and clinical prognosis factors in surgical patients.

## METHODS

It is an observational and longitudinal study realized in the General Surgery Unit of Asa Norte Regional Hospital , Brasília, DF, Brazil, between November of 2012 and April of 2013. This study was approved by the Ethics Committee in Research with Human Beings of the Federal District Department of Health under the statement nº 144.850 and Presentation Certificate for Ethics Appreciation n^o^ 06025012.7.0000.5553 on 11/12/2012.

The inclusion criteria were: patients at the age of 20 years old or more, and less than 60 years old of both genders, submitted to surgery in the gastrointestinal tract who were hospitalized for a minimum period of three days after the surgery for the realization of biochemical exams. The excluded patients were those who presented any physical disability, pregnant patients, nursing mothers, patients confined to bed, those with diagnoses of any comorbidity (diabetes mellitus, dyslipidemia, AIDS), those who were using immunosuppressive medication, patients hospitalized for bariatric surgery, and those coming from the urology sector, besides the non-communicative individuals or those who were unable to understand the Informed Consent Form.

For the sample, it was considered a population of 80 individuals, which consists of the average of the monthly hospitalizations at the General Surgery Unit and a prevalence of 48.1% of hospital malnutrition, based on the results of the Brazilian Survey of Hospital Nutritional Assessment (IBRANUTRI) 9 . It was considered an error of ±5% for a level of significance of 95%, which resulted in a minimum sample of 67 patients. The program OpenEpi, version 3.01 8 , was used for statistics. All of the patients were monitored until the hospital discharge.

After the selection, all of the participants went through nutritional triage and did biochemical exams pre and postoperative for the observation of biochemical alterations and investigation of surgical complications.

The assessment of the patients was done through semi-structured and pre-codified survey, which had socioeconomic and clinical questions, and through the Subjective Global Assessment (SGA).

The SGA consists of a model of standardized questionnaire used for the investigation of clinical history and physical examination of the patient. This questionnaire was proposed by Baker et al. 2 . It is a simple, cheap, and fast method that can be used on patient's bed. It has practical and relevant questions about the clinical history and physical examination of the patient, which allows the classification in three categories: A=nourished; B=mildly malnourished (or suspected of malnutrition); and C=severely malnourished 10 24 .

The anthropometric variables used were: weight, height, body mass index (BMI), triceps skinfold thickness (TST), arm circumference (AC), and arm muscle circumference (AMC). The non-dominant side of the patient was used for the measurements of the circumference and TST 10 .

The BMI was calculated through the division of the weight (kg) by the squared height (m). The values of the BMI were classified in: <18.5 kg/m^2^ (underweight); 18.5 to 24.9 kg/m^2^ (normal); 25 to 29.9 kg/m^2^ (overweight); and ≥30 kg/m^2^ (obesity) according to the World Health Organization 29 .

For the measurement of the triceps skinfold thickness, the researchers used Cescorf® adipometer with a reading amplitude of 88 mm. The results were compared to the standard values established by Frisancho 11 . The classification of the nutritional status was realized according to the parameters established by Blackburn and Thornton 4 .

The arm circumference (AC), triceps skinfold thickness (TST), and arm muscle circumference (AMC) were used to diagnose alterations in the body muscle mass and the protein nutritional status 4 . The result was compared to the reference values in percentiles according to Frisancho 11 . The AMC was classified according to the values described by Jellife 14 .

For the laboratorial assessment, the researchers used the pre-operative exams required in the triage of the patient for the realization of the surgery. The exams were done by the staff of the pharmacy of the hospital laboratory of clinical analysis. The indicators analyzed were the values of serum albumin and the total lymphocyte count.

For the classification of the nutritional status according to albumin, the following reference values were adopted: >3.5 g/dl (nourished), 3.0 a 3.5 g/dl (mild malnutrition), 2.4 a 2.9 g/dl (moderate malnutrition), and <2.4 g/dl(severe malnutrition) 18 . 

The total lymphocyte count was calculated through a leucogram using the lymphocyte percentage and the value of the lymphocytes (ml). The cut-off points used for the classification of the nutritional status (immunologic depletion) according to the TLC were: >2000 cells/m^3^ (normal), 1,200 to 2,000 cells/m^3^ (mild depletion), 800 to 1,199 cells/m^3^ (moderate depletion), and < 800 cells/m^3^ (severe depletion) 6 .

The data was processed in Excel software and analyzed in the statistics program SPSS, version 19.0 for Windows. The normality test of Kolmogorov-Smirnov was used and, due to the obtained results, Spearman correlation was also used. Pearsons' correlation test verified the degree of correlation between the variables for the diagnostic without categories. There were analyses through Kappa statistics, odds ratio, univariate logistic regression, and chi-square test for the purpose of comparison between the several methods, considering the classifications: nourished, well nourished, and normal as nourished individuals, and mild depletion, moderate depletion, and severe depletion as malnourished individuals. It was adopted p<0.05 as significant.

## RESULTS

The sample was composed of 69 patients, 50.7% (n=35) were female, at an average age of 43±9.8 years old. A little over half of the interviewees 50.7% (n=35) had completed middle school, and 49.3% (n=34) receive up to two minimum wages per month.

When interrogated about recent and previous hospitalization for the realization of surgeries and if there were any postoperative complications during this time, 34.8% (n=24) reported recent hospitalization and from those, 11.5% reported postoperative complications; 60.9% (n=42) considered their health statuses as inadequate at the moment of the interview.

Regarding the clinical and anthropometric characteristics of the sample, among the clinical data, the average of albumin was within the normal levels. However, there was mild depletion for TLC and a great variation regarding the minimum and maximum encountered values ( Table 1 ).

**TABLE 1:** Clinical and anthropometric characteristics of the patients (n=69)

Variable	Average	Median	Standard deviation	[Minimum - Maximum]
Current weight (kg)	64.8	63.9	12.8	43 - 106
Height (m)	1.63	1.65	0.1	1.38 - 1.83
Age (years)	43.6	46	9.8	19 - 59
Albumin (g/dL)	3.61	3.7	0.69	1.7 - 5.4
TLC (cells/m³)	1,552.5	1,452	921	105 - 4269
AC (cm)	29.0	28.5	4.04	18.1 - 40.1
AC (%)	93.45	91.9	14.4	55.1 - 153.2
TST (mm)	17.44	17	8.39	3 - 40
TST (%)	97.0	96	36.7	25 - 233
AMC (cm)	23.5	23.9	3.2	15.3 - 32.7
AMC (%)	94.9	92.0	15.2	58.8 - 139.1

TLC=total lymphocyte count; AC=arm circumference; AC (%)=% adequacy of the arm circumference; TST=triceps skinfold thickness; TST (%)=% adequacy of the triceps skinfold thickness; AMC=arm muscle circumference; AMC (%)=% adequacy of the arm muscle circumference

The diagnosis of the nutritional status through the objective and subjective methods showed that the prevalence of malnutrition was 40.6% (n=28) through albumin, 73.9% (n=51) through the TLC, 49.2% (n=34) through the SGA, and only 7.2% (n=05) through the BMI ( Table 2 ).

**TABLE 2:** Assessment of the nutritional status through subjective and objective methods of patients hospitalized (n=69)

Variables	n	PFD (%)	CI 95%
BMI
Overweight + obesity	29	42.0	0.31-0.53
Normal	35	50.7	0.39-0.62
Malnutrition	5	7.2	0.03-0.15
SGA
Nourished	35	50.7	0.39-0.62
Mildly malnourished (or suspected of malnutrition);	23	33.3	0.23-0.45
Severely malnourished	11	15.9	0.09-0.26
ALBUMIN
Nourished	41	59.4	0.47-0.70
Malnutrition	28	40.6	0.23-0.68
TLC
Nourished	18	26.1	0.17-0.37
Malnutrition	51	73.9	0.48-1.05
% AC
Overweight+obesity	6	8.7	0.04-0.17
Normal	37	57.6	0.41-0.64
Malnutrition	26	37.7	0.20-0.64
% TST	
Overweight+obesity	18	26.1	0.17-0.37
Normal	25	36.2	0.25-0.48
Malnutrition	26	37.7	0.20-0.64
% AMC
Overweight+obesity	13	18.8	0.11-0.29
Normal	29	42.0	0.31-0.53
Malnutrition	27	39.1	0.21-0.69

n=number of patients; PFD(%)=percentage frequency distribution; BMI=body mass index; CI=confidence interval; SGA=subjective global assessment; TLC=total lymphocyte count; %AC=% adequacy of the arm circumference; %TST=% adequacy of the triceps skinfold thickness; %AMC=% adequacy of the arm muscle circumference

The concordance of the diagnoses between albumin and the TLC with the values of the BMI and the SGA showed that none of the two methods presented good concordance with the BMI (k=-0.002 and k=0.05 respectively), and only albumin presented moderate concordance (k=0.47) when compared to the SGA.

When trying to verify the degree of the correlation between the variables for the diagnoses (albumin, TLC, BMI, and SGA), it was observed that albumin (r=0.52; p< 0.01) presented positive and significant correlation with the scores of the SGA. When compared to the BMI, neither albumin (r= 0.06; p=0.59) nor the TLC (r=-0.15; p=0.9) presented significant results.

When evaluating the influence of the values of the TLC and albumin regarding the risk of postoperative complications after univariate logistic regression, there was an association between albumin (p=0.01) and the TLC (p=0.02).

When comparing the results of the SGA with the risk of complications, it was determined that as SGA scores got higher, the risk of postoperative complications increased as well (p<0.001).

Regarding the anthropometric methods evaluated, only the percentage of adequacy of triceps skinfold thickness proved to be a good predictor of nutritional risk for surgical patients (p<0.02). 

## DISCUSSION

Protein-energy malnutrition is very common among hospitalized patients, and it is a problem caused by several factors, from determiners related to the clinical condition until the monitoring of the hospitalized patient 16 .

The prevalence of malnutrition in this study was high for both the objective and subjective methods, except the BMI. Leandro-Merhi et al. 15 also found that almost half of the patients (45.4%) were classified as normal according to the BMI when assessing the postoperative nutritional risk of the patients.

Although the BMI is an internationally accepted method for the classification of nutritional status, it does not assess the body composition and has several limitations. A few studies mention the limitation of the BMI as a method that does not provide accurate information about weight alterations related to an increase in the lean body mass or body fat, it has low sensibility to detect individuals with increased body fat, and it does not consider gender and ethnicities 19 23 . The adoption of the BMI as the only standard for the classification of nutritional status may lead to inaccurate assessments, incorrect diagnosis, and possible erroneous interventions 15 .

It is important to be attentive to the nutritional states of the patients who are candidates to surgical procedures in order to identify those who may possibly develop malnutrition or those who are already hospitalized with this nutritional status.

The results of this study show that among the anthropometric measurements, only the percentage of adequacy of triceps skinfold thickness was a good predictor of the risk of surgical complications. The TST is a simple, cheap, safe, and non-invasive method that may be useful in the assessment of long-term changes in the reservoirs of subcutaneous adipose tissue, and its variations can be monitored during the period of the hospitalization 26 .

In the assessed patients, the SGA, the total lymphocyte count, and albumin were good nutritional predictors of postoperative complications, considering that as the SGA scores got higher, the risk of complications also increased, and as the parameters of albumin and TLC increased, the risk of surgical complications reduced. Considering the obtained values, albumin proved to be a better predictor of the risk than the total lymphocyte count in the assessed sample.

The reduction in the anthropometric and biochemical markers, such as albumin, transferrin, and the TLC, influence in the clinical condition of the patient, since the greater the depletion, the greater the detected pre-surgical risk 26 .

In studies conducted by Valero et al. 27 was found a positive association with the values of serum albumin and lymphocytes in the assessment of nutritional status. The researchers assessed 135 patients and detected that 0.7% of the sample presented the number of lymphocytes lower than 800/ul and 37.5% presented serum albumin lower than 3.5 g/dl. The patients with lower values of lymphocytes and albumin had worse nutritional status.

Mandroño et al. 17 conducted a study with 101 patients at University Hospital in Spain evaluating the relation between the serum levels of total cholesterol (TC), total albumin, and the TLC with different methods of nutritional assessment. The results determined that the three parameters evaluated in the study had significant increased sensibility, showing that, according to the standard value that was established, lower the values of the parameters, worse was nutritional status of the assessed patient.

To have a better approach of the hospitalized patient, the American Society for Parenteral and Enteral Nutrition - ASPEN 1 recommends as a way to diagnose malnutrition, besides the subjective nutritional assessment, a combination of clinical, biochemical, and anthropometric parameters. The institution highlights that the assessment of the patient as a whole is important in order to detect the nutritional risk, considering the increased risk of morbimortality due to the depleted nutritional status 3 . Besides the diagnosis of malnutrition, it is essential to evaluate the risk of nutritional deterioration in patients with conditions associated with nutritional problems 5 .

In spite of the limitations of the biochemical parameters, albumin is one of the most used variables in the clinical practice, and there has been demonstrated positive association between hypoalbuminemia and complications in hospitalized patients 25 . This parameter is directly associated with nutritional status, more specifically with protein-energy malnutrition, and with the severity of the disease, being an indicator of risk in surgical patients with a severe condition, patients in intensive care unit, patients with inflammatory diseases or traumatic brain injury 7 22 . 

It is observed that the immunological alterations, such as the reduction of the TLC, increase the frequency and the severity of the infection. This is responsible for a great part of the morbity and mortality associated with nutrition. The total lymphocyte count has been suggested as a useful indicator of nutritional status and it should be also considered in the clinical environment 1 .

It is important to mention a few limitations of this study, such as the size of the sample and the patients subjected to different types of gastrointestinal surgeries with higher/lower degrees of severity. More studies are necessary in different populations in order for these parameters to be validated as a means of early detection of morbimortality in surgical patients.

## CONCLUSION

Subjective and objective methods detected a high prevalence of malnutrition, except the BMI. The biochemical values of albumin, total lymphocyte count, and the classification of the SGA and %TST were correlated as predictors of risk of postoperative complications. Albumin and the TLC together with other ways of assessment of nutritional status, mainly the SGA and the %TST, are useful methods to better identify nutritional status, making it possible to identify the nutritional risks and possibly as indicators of postoperative complications.
